# MRI-guided risk stratification for neoadjuvant immunotherapy in rectal cancer

**DOI:** 10.3389/fimmu.2026.1782231

**Published:** 2026-05-29

**Authors:** Jiali Zhang, Feng Tian, Yue Shang, Honghai Dai, Shuo Zhang, Bing Kang, Jiaxiang Xin, Ximing Wang, Changqing Jing, Cong Sun

**Affiliations:** 1 Department of Radiology, Shandong Provincial Hospital Affiliated to Shandong First Medical University, Jinan, China; 2 Shandong First Medical University (Shandong Academy of Medical Sciences), Jinan, Shandong, China; 3 Department of Gastrointestinal Surgery, Shandong Provincial Hospital Affiliated to Shandong First Medical University, Jinan, China; 4 Tumor Research and Treatment Center, Shandong Provincial Hospital Affiliated to Shandong First Medical University, Jinan, China; 5 Magnetic Resonance (MR) Research Collaboration, Siemens Healthineers Ltd., Shanghai, China

**Keywords:** magnetic resonance imaging, neoadjuvant immunotherapy, pathological complete response, rectal cancer, risk stratification

## Abstract

**Background:**

Neoadjuvant therapy improves local control and tumor downstaging in locally advanced rectal cancer (LARC) and is now the standard of care. However, reliable tools for identifying patients who may show different response patterns to neoadjuvant immunotherapy-containing treatment, particularly MRI-based risk stratification systems, remain limited.

**Materials and methods:**

This retrospective study included 135 patients with locally advanced rectal cancer, who were classified into a neoadjuvant immunotherapy plus chemoradiotherapy group (nICRT, n = 43) and a neoadjuvant chemoradiotherapy group (nCRT, n = 92). The nICRT group received short-course radiotherapy plus CAPOX and sintilimab, whereas the nCRT group received either short-course radiotherapy plus CAPOX or long-course chemoradiotherapy plus CAPOX. All patients underwent baseline pelvic MRI, including high-resolution T2-weighted, diffusion-weighted, and T1-weighted sequences. Evaluated variables included mrT stage, mrN stage, mrEMVI status, mrMRF involvement, tumor length, and clinical laboratory indices. Interobserver agreement was assessed using Cohen’s kappa statistics, and factors associated with pathological complete response (pCR) were analyzed using group comparisons and logistic regression analyses. Fisher’s exact test was used for subgroup comparisons. Receiver operating characteristic analysis was performed to evaluate predictive performance, and bootstrap resampling was used for internal validation.

**Results:**

An MRI-based assessment using mrEMVI, mrMRF, and tumor length ≥ 5 cm yielded a three-factor risk score that predicted pCR with an AUC of 0.835. In the MRI high-risk group (2–3 points), pCR rates were higher with nICRT than with nCRT (7/19, 36.8% vs. 7/60, 11.7%, P = 0.033), whereas in the low-risk group (0–1 points) the difference was not significant (19/24, 79.2% vs. 21/32, 65.6%, P = 0.373). In a sensitivity analysis restricted to the short-course SPRING-01 cohort, the direction of the association remained similar, although statistical significance was not retained.

**Conclusion:**

In MRI-defined high-risk patients, nICRT was associated with a higher pCR rate than nCRT. These findings suggest that pretreatment MRI-based stratification may help identify clinically relevant subgroups with different response patterns to intensified neoadjuvant treatment. However, the results should be considered exploratory and require prospective validation before clinical application.

## 1 Introduction

Locally advanced rectal cancer (LARC) remains a major clinical challenge because of its high risk of recurrence and unsatisfactory long-term survival (1). The current standard treatment is neoadjuvant chemoradiotherapy (nCRT) followed by total mesorectal excision (TME), yet the pathological complete response (pCR) rate is only 15%–27% (2), highlighting the need for more effective treatment strategies.

In recent years, immune checkpoint inhibitors (ICIs) have demonstrated substantial clinical benefit across multiple malignancies, including colorectal cancer. Notably, the efficacy of ICIs in colorectal cancer is influenced by mismatch repair (MMR) and microsatellite instability (MSI) status, with the greatest benefit observed in mismatch repair-deficient/microsatellite instability-high (dMMR/MSI-H) tumors (3–5). As most LARC cases are proficient mismatch repair/microsatellite stable (pMMR/MSS) and generally less immunogenic, the use of immunotherapy in unselected rectal cancer remains investigational rather than standard of care (6). Nevertheless, radiotherapy and chemotherapy may modulate the tumor microenvironment and enhance immune priming, providing a rationale for combining immune checkpoint inhibitors with chemoradiotherapy (7–9). Early-phase studies, including TORCH and SPRING-01, have suggested that such combinations may improve tumor regression and pCR in predominantly pMMR/MSS LARC, with pCR rates of 50–60% (10, 11). Although these findings are encouraging, treatment responses remain heterogeneous, and reliable criteria for identifying patients who may be more likely to respond to these intensified neoadjuvant regimens are still lacking.

Magnetic resonance imaging (MRI) plays a pivotal role in the management of rectal cancer and is now routinely used in LARC to assess key tumor characteristics, including extramural venous invasion (EMVI), mesorectal fascia (MRF) involvement, and tumor burden, which are closely related to recurrence risk and survival (12–15). However, whether these pretreatment MRI features can help identify clinically relevant subgroups with differential response to neoadjuvant immunotherapy plus chemoradiotherapy (nICRT) remains largely unexplored. Therefore, this study aimed to develop a pretreatment MRI-based risk stratification system for patients with LARC and to investigate, in a hypothesis-generating manner, whether MRI-defined risk subgroups were associated with different pCR patterns under nICRT compared with conventional nCRT.

## 2 Materials and methods

This retrospective study was approved by the institutional review board of our hospital, which waived the requirement for written informed consent owing to the retrospective design.

### 2.1 Patients

This retrospective study initially reviewed 503 patients with LARC, including 98 enrolled in the SPRING-01 trial and 405 treated with neoadjuvant chemoradiotherapy followed by surgery between January 2020 and December 2024. Among the 98 patients from SPRING-01, 13 were excluded because of incomplete pretreatment clinical data, missing or poor-quality baseline MRI, incomplete neoadjuvant treatment, a history of other malignant tumors or prior metastasis, mucinous adenocarcinoma, or failure to undergo TME surgery. The remaining 85 patients comprised 43 treated with short-course radiotherapy plus CAPOX and sintilimab and 42 treated with short-course radiotherapy plus CAPOX alone.

Among the 405 patients treated outside the trial, the inclusion criteria were as follows: histologically confirmed rectal adenocarcinoma, available baseline MRI and clinical laboratory results, receipt of long-course chemoradiotherapy followed by 2–4 cycles of CAPOX, and availability of postoperative pathological assessment. Patients with poor-quality MRI or a history of other malignancies or prior metastasis were excluded, yielding 50 additional patients treated with long-course chemoradiotherapy eligible for inclusion.

**Figure 1:**
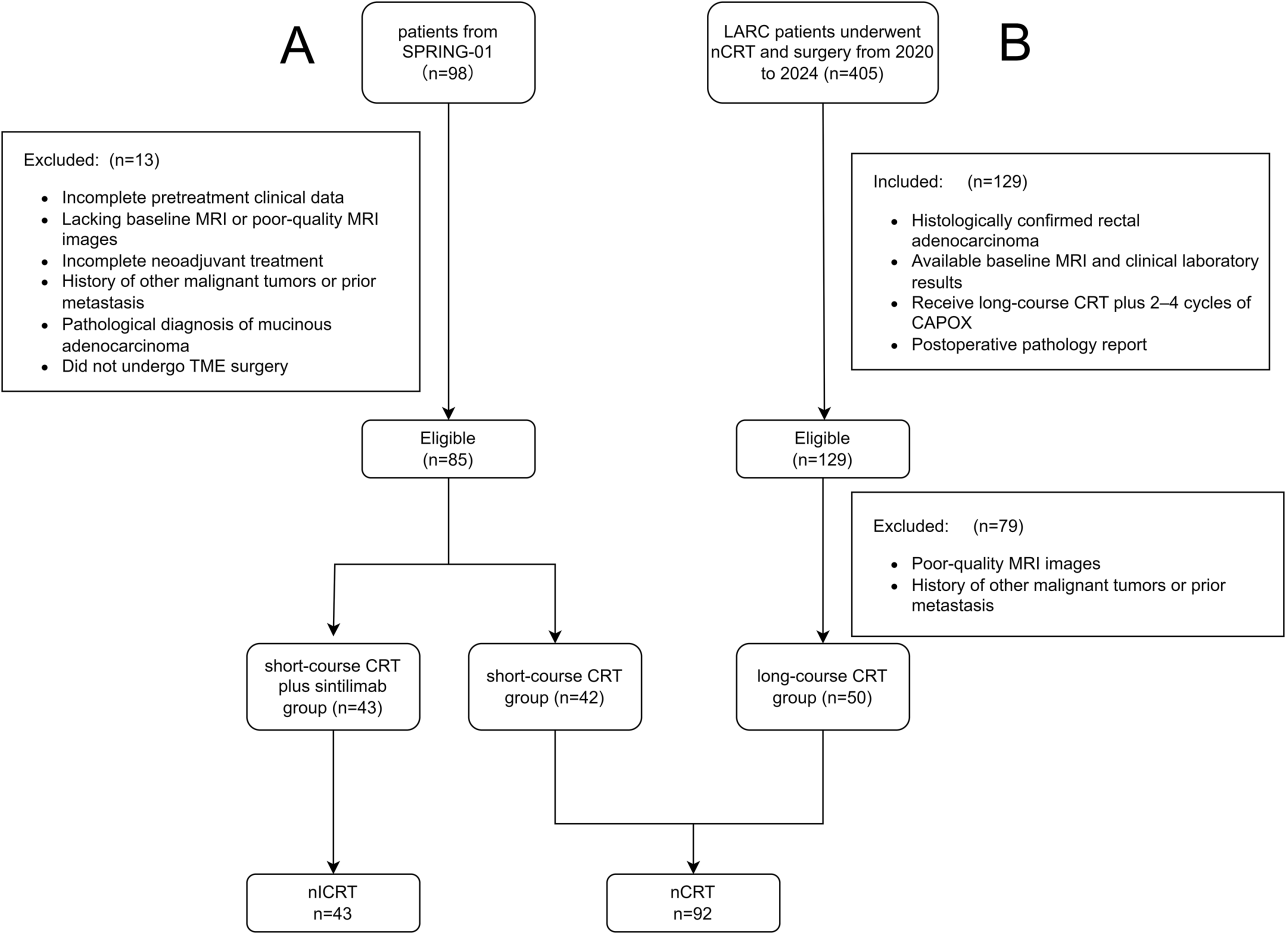
Flowchart of patient selection from **(A)** the SPRING-01 trial cohort and **(B)** the clinical cohort of patients with locally advanced rectal cancer (LARC). MRI, magnetic resonance imaging; CRT, chemoradiotherapy; CAPOX, capecitabine plus oxaliplatin; TME, total mesorectal excision.

In total, 135 patients were included and classified into a nICRT group (n = 43) and a nCRT group (n = 92; 42 receiving short-course treatment and 50 receiving long-course treatment) (**Figure 1**).

### 2.2 Treatment protocols

Three distinct neoadjuvant treatment regimens were used in this study. Patients in the nICRT group (n = 43) received short-course radiotherapy (5 × 5 Gy over 5 consecutive days), followed by six 3-week cycles of CAPOX chemotherapy, with intravenous sintilimab (200 mg) administered on day 1 of each cycle. Patients in the nCRT group received one of two conventional regimens: (1) short-course radiotherapy followed by six cycles of CAPOX without sintilimab; or (2) long-course radiotherapy to a total dose of 45–50.4 Gy delivered in 25–28 fractions (1.8–2.0 Gy per fraction, 5 fractions per week), with concurrent capecitabine on radiotherapy days, followed by 2–4 cycles of CAPOX.

### 2.3 MR examination and image analysis

All patients underwent pretreatment pelvic MRI on a 3.0-T scanner from two manufacturers (Siemens Healthcare, Erlangen, Germany, and Philips Healthcare, Best, the Netherlands) using a phased-array body coil in the supine position, without routine bowel preparation or administration of antiperistaltic agents. The MRI protocol included high-resolution T2-weighted turbo spin-echo sequences in the sagittal, axial oblique (perpendicular to the tumor axis), and coronal planes. Axial diffusion-weighted imaging (DWI) was performed with b values of 0 and 1000 s/mm², and corresponding apparent diffusion coefficient (ADC) maps were automatically generated from the DWI data. Axial T1-weighted imaging was acquired before and, when available, after intravenous contrast administration. Detailed acquisition parameters are provided in **Supplementary Table S1**.

MRI images were independently reviewed by two radiologists with 5 and 20 years of experience in rectal MRI, blinded to clinical and pathological outcomes. The following parameters were recorded according to established guidelines and previous literature: baseline mrT and mrN stage according to the 8th edition of the American Joint Committee on Cancer (AJCC) staging system; MRF involvement and EMVI according to ESGAR criteria (16, 17); tumor length measured along the longest axis on sagittal T2-weighted images (T2WI); circumferential extent defined as the proportion of bowel circumference involved on axial T2WI; distance to the anal verge measured along the bowel lumen; and maximum tumor thickness as the greatest radial diameter. Specifically, mrEMVI status was assessed on axial high-resolution T2WI using the 5-point grading system described by Smith et al. (14), with grades 3 and 4 considered EMVI-positive. mrMRF involvement was defined as a minimum distance of < 1 mm between the tumor, tumor deposits, or involved lymph nodes and the mesorectal fascia. Tumor length was measured on sagittal T2WI as the maximum craniocaudal extent of the tumor and was dichotomized at 5 cm based on the distribution of data and prior literature (18, 19). Any discrepancies between the two readers were resolved by consensus after joint review.

### 2.4 Collection of laboratory variables

Pretreatment laboratory variables obtained within 1 week before treatment initiation were collected from medical records, including carcinoembryonic antigen (CEA), platelet count (PLT), and inflammatory indices. CEA was categorized as < 5 or ≥ 5 ng/mL. Inflammatory indices included neutrophil-to-lymphocyte ratio (NLR), platelet-to-lymphocyte ratio (PLR), and lymphocyte-to-monocyte ratio (LMR), which were calculated from baseline blood counts.

### 2.5 Pathological and molecular assessment

Each surgical specimen was independently reviewed in a blinded manner by two pathologists with 10 and 25 years of experience, respectively. pCR was defined according to the 8th edition of the AJCC as the absence of residual viable tumor cells in both the primary tumor site and regional lymph nodes in the resected specimen (yT0N0); non-pCR was defined as any residual viable tumor in the primary tumor site and/or lymph nodes. MMR status was assessed by immunohistochemistry (IHC) on pretreatment biopsy or surgical resection specimens using antibodies against four MMR proteins: MLH1, MSH2, MSH6, and PMS2. Loss of nuclear expression of one or more MMR proteins was classified as dMMR, while retained expression of all four proteins was classified as pMMR. Any discrepancies between observers were resolved by consensus after joint review.

### 2.6 Statistical analysis

Inter-reader agreement between the two radiologists was assessed using Cohen’s kappa statistic for dichotomized imaging variables. Baseline characteristics were compared between treatment groups using the chi-square test or Fisher’s exact test for categorical variables, and the Student’s t-test or Mann–Whitney U test for continuous variables, as appropriate.

Variables associated with pCR were first explored by comparing baseline characteristics between the pCR and non-pCR groups. Candidate variables were assessed in univariable logistic regression, and variables with P < 0.05 were entered into the multivariable logistic regression model to identify independent factors associated with pCR status. Odds ratios (ORs) and 95% confidence intervals (CIs) were reported.

A composite MRI-based risk score was constructed by summing the number of independent MRI predictors present for each patient (range 0–3; one point per factor). The optimal cut-off for dichotomizing patients into low-risk and high-risk groups was determined using Youden’s index. Differences in pCR rates between the nCRT and nICRT groups within each risk category were compared using Fisher’s exact test based on 2×2 contingency tables. To assess the robustness of the subgroup findings, the pCR comparison was repeated in the randomized SPRING-01 subset according to MRI-defined risk status. Treatment effects were visualized using forest plots with corresponding ORs and 95% CIs. ORs, 95% CIs, and P values were estimated using univariable logistic regression within each subgroup, with nCRT as the reference category. ORs > 1 indicate a higher likelihood of achieving pCR in the nICRT group than in the nCRT group.

Discriminative performance was evaluated using the area under the receiver operating characteristic curve (AUC), and comparisons between the composite score and individual MRI parameters were performed using the DeLong test. Internal validation of the composite score was performed using 2,000 bootstrap resamples to estimate the optimism-corrected AUC, calibration slope, and calibration intercept. All statistical analyses were performed using R software (version 4.4.3; R Foundation for Statistical Computing, Vienna, Austria), and a two-sided P value < 0.05 was considered statistically significant.

## 3 Results

### 3.1 Baseline characteristics according to treatment group

Baseline characteristics of the study cohort are presented in **Table 1**. A total of 135 patients were included, of whom 92 received nCRT and 43 received nICRT. Overall, most clinicoradiological characteristics were comparable between the two groups, although significant differences were observed in age and tumor length. As expected, treatment composition differed by design, because the nCRT cohort included both short-course (n = 42) and long-course radiotherapy (n = 50), whereas the nICRT cohort (n = 43) consisted exclusively of short-course radiotherapy plus immunotherapy. MMR status was available in 98 of 135 patients (72.6%), and most evaluable patients were pMMR.

**Table 1:** Baseline characteristics according to treatment group.

Variable	Total (n = 135)	nCRT (n = 92)	nICRT (n = 43)	P value
Sex				0.52
Male	93 (68.9%)	65 (70.7%)	28 (65.1%)	
Female	42 (31.1%)	27 (29.3%)	15 (34.9%)	
Age (years)				0.014
< 60	64 (47.4%)	37 (40.2%)	27 (62.8%)	
≥ 60	71 (52.6%)	55 (59.8%)	16 (37.2%)	
mrT stage				0.32
T2	1 (0.7%)	0 (0.0%)	1 (2.3%)	
T3	89 (65.9%)	62 (67.4%)	27 (62.8%)	
T4	45 (33.3%)	30 (32.6%)	15 (34.9%)	
mrN stage				0.27
N0	10 (7.4%)	5 (5.4%)	5 (11.6%)	
N1	61 (45.2%)	45 (48.9%)	16 (37.2%)	
N2	64 (47.4%)	42 (45.7%)	22 (51.2%)	
mrEMVI status				0.98
Negative	41 (30.4%)	28 (30.4%)	13 (30.2%)	
Positive	94 (69.6%)	64 (69.6%)	30 (69.8%)	
mrMRF involvement				0.26
Negative	69 (51.1%)	44 (47.8%)	25 (58.1%)	
Positive	66 (48.9%)	48 (52.2%)	18 (41.9%)	
Tumor length (cm)				0.01
< 5	70 (51.9%)	41 (44.6%)	29 (67.4%)	
≥ 5	65 (48.1%)	51 (55.4%)	14 (32.6%)	
Distance to the anal verge (cm)				0.50
≤ 5	54 (40.0%)	35 (38.0%)	19 (44.2%)	
> 5	81 (60.0%)	57 (62.0%)	24 (55.8%)	
Tumor circumferential extent				0.14
< 1/4	3 (2.2%)	3 (3.3%)	0 (0.0%)	
1/4 ≤ x < 1/2	10 (7.4%)	9 (9.8%)	1 (2.3%)	
1/2 ≤ x < 3/4	25 (18.5%)	19 (20.7%)	6 (14.0%)	
3/4 ≤ x ≤ 1	97 (71.9%)	61 (66.3%)	36 (83.7%)	
CEA (ng/mL)				0.86
< 5	80 (59.3%)	55 (59.8%)	25 (58.1%)	
≥ 5	43 (31.9%)	37 (40.2%)	18 (41.9%)	
MMR status				0.26
dMMR	2 (1.5%)	1 (1.1%)	1 (2.3%)	
pMMR	96 (71.1%)	68 (73.9%)	28 (65.1%)	
Unknown	37 (27.4%)	23 (25.0%)	14 (32.6%)	
Neoadjuvant regimen				< 0.001
SCRT + CAPOX + sintilimab	43 (31.9%)	0 (0.0%)	43 (100.0%)	
SCRT + CAPOX	42 (31.1%)	42 (45.7%)	0 (0.0%)	
LCRT + CAPOX	50 (37.0%)	50 (54.3%)	0 (0.0%)	

Data are presented as n (%), unless otherwise indicated. Percentages are column percentages. P values were calculated using the chi-square test or Fisher’s exact test, as appropriate. nCRT = neoadjuvant chemoradiotherapy; nICRT, neoadjuvant immunotherapy plus chemoradiotherapy; mrEMVI, magnetic resonance imaging-detected extramural venous invasion; mrMRF, magnetic resonance imaging-detected mesorectal fascia involvement; MMR, mismatch repair; dMMR, deficient mismatch repair; pMMR, proficient mismatch repair; SCRT, short-course radiotherapy; LCRT, long-course chemoradiotherapy; CAPOX, capecitabine plus oxaliplatin; CEA, carcinoembryonic antigen.

As summarized in **Supplementary Table S2**, baseline demographic and clinical characteristics were broadly comparable between the pCR and non-pCR groups. With respect to MRI features, mrEMVI positivity, mrMRF involvement, and tumor length ≥ 5 cm were significantly less frequent in the pCR group than in the non-pCR group (mrEMVI: 42.6% vs. 87.7%, P < 0.001; mrMRF: 24.1% vs. 65.4%, P < 0.001; tumor length ≥ 5 cm: 24.1% vs. 64.2%, P < 0.001), whereas mrT stage, mrN stage, distance to the anal verge, tumor circumferential extent, and maximum tumor thickness showed no significant association with pCR status. In addition, the mean pretreatment platelet count was lower in patients achieving pCR compared with those without pCR (219.5 ± 69.3 vs. 261.2 ± 84.1, P = 0.003), while other laboratory indices showed no significant between-group differences.

### 3.2 Univariable and multivariable analysis of factors associated with non-pCR

In univariable analysis, the mrEMVI positivity (OR, 9.57; 95% CI, 4.07–22.48; P < 0.001), mrMRF involvement (OR, 5.97; 95% CI, 2.75–12.94; P < 0.001), tumor length ≥ 5 cm (OR, 5.66; 95% CI, 2.61–12.24; P < 0.001), and platelet count (OR, 1.01; 95% CI, 1.00–1.01; P = 0.005) were significantly associated with non-pCR. In multivariable analysis, mrEMVI positivity (OR, 8.64; 95% CI, 3.21–23.27; P < 0.001), mrMRF involvement (OR, 4.03; 95% CI, 1.58–10.27; P = 0.004), and tumor length ≥ 5 cm (OR, 3.26; 95% CI, 1.32–8.05; P = 0.011) remained independent predictors (**Table 2**).

**Table 2:** Univariable and multivariable analysis of factors associated with non-pCR.

Variables	Univariate analysis		Multivariate analysis	
	OR (95% CI)	*P* value	OR (95% CI)	*P* value
mrEMVI positivity	9.57 (4.07–22.48)	< 0.001	8.64 (3.21–23.27)	< 0.001
mrMRF involvement	5.97 (2.75–12.94)	< 0.001	4.03 (1.58–10.27)	0.004
Tumor length ≥ 5 cm	5.66 (2.61–12.24)	< 0.001	3.26 (1.32–8.05)	0.011
PLT	1.01 (1.00–1.01)	0.005	1.00 (0.99–1.01)	0.217

ORs and 95% confidence intervals (CIs) are shown. Variables with P < 0.05 in univariable analysis were entered into the multivariable logistic regression model. OR > 1 indicates a higher likelihood of non-pCR. mrEMVI, magnetic resonance imaging-detected extramural venous invasion; mrMRF, magnetic resonance imaging-detected mesorectal fascia involvement; PLT, platelet count.

### 3.3 Interobserver agreement

Interobserver agreement for the MRI-derived risk factors ranged from substantial to almost perfect, with κ values of 0.787 for mrEMVI (95% CI, 0.67–0.90), 0.733 for mrMRF (95% CI, 0.62–0.85), and 0.881 for tumor length (95% CI, 0.80–0.96), all P < 0.001 (**Supplementary Table S3**).

### 3.4 Construction and discrimination of the MRI-based risk score

**Figure 2:**
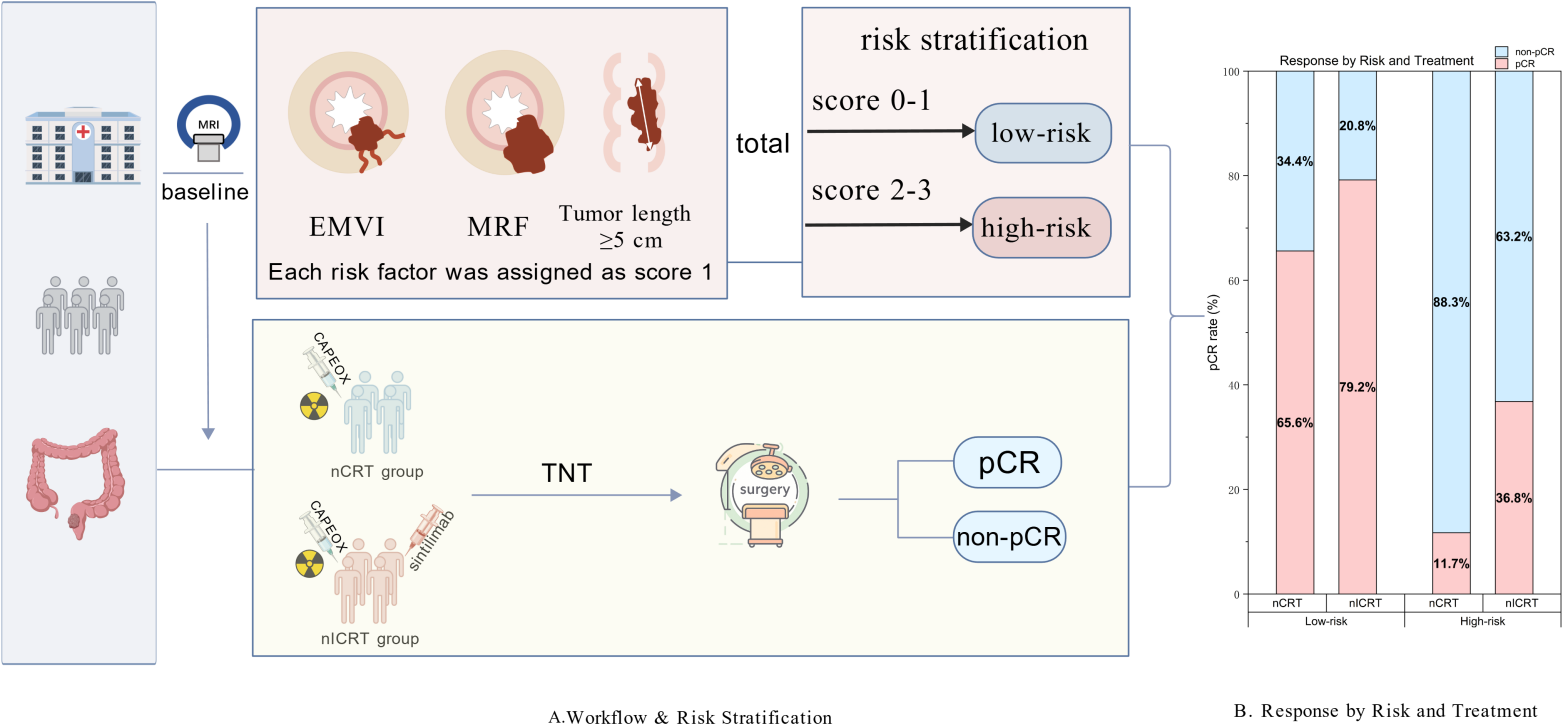
Workflow of MRI-based risk stratification and pCR rates by risk group and treatment. **(A)** Baseline MRI-based risk stratification. mrEMVI, mrMRF, and tumor length ≥ 5 cm were each assigned 1 point; total scores of 0–1 defined the low-risk group, and scores of 2–3 defined the high-risk group. Patients underwent total neoadjuvant therapy (TNT) with either nCRT or nICRT before surgery. **(B)** Stacked bar chart showing the proportions of pCR (red) and non-pCR (blue) according to risk group and treatment. Percentages are shown within the bars; corresponding patient numbers are provided in **Table 3**. mrEMVI, magnetic resonance imaging-detected extramural venous invasion; mrMRF, magnetic resonance imaging-detected mesorectal fascia involvement; TNT, total neoadjuvant therapy; nCRT, neoadjuvant chemoradiotherapy; nICRT, adjuvant immunotherapy plus chemoradiotherapy; pCR, pathological complete response.

An MRI-based risk stratification system was constructed using three MRI risk factors (**Figure 2A**): mrEMVI positivity, mrMRF involvement, and tumor length ≥ 5 cm. Based on Youden’s index, a cut-off of 1.5 for the total score provided the greatest separation in pCR rates, and patients were therefore categorized as low risk (0–1 points) or high risk (2–3 points). Among the 135 patients, 56 (41.5%) were classified as low risk and 79 (58.5%) as high risk (**Figure 2B**). This MRI-based scoring system significantly distinguished between patients with and without pCR across treatment groups (**Supplementary Table S4**).

In the overall cohort, the association between treatment group and pCR appeared to differ according to MRI-defined risk status. In the high-risk group, the pCR rate was higher in the nICRT group than in the nCRT group (7/19, 36.8% vs. 7/60, 11.7%; P = 0.033), whereas no significant benefit was observed in the low-risk group (19/24, 79.2% vs. 21/32, 65.6%; P = 0.373) (**Table 3A**).

**Table 3:** pCR rates by treatment group according to the MRI-based risk score in the overall cohort and sensitivity subset.

Panel A. overall cohort				
Group	pCR, n (%)	non-pCR, n (%)	Total, n	P value
Low-risk				0.373
nCRT	21 (65.6)	11 (34.4)	32	
nICRT	19 (79.2)	5 (20.8)	24	
High-risk				0.033
nCRT	7 (11.7)	53 (88.3)	60	
nICRT	7 (36.8)	12 (63.2)	19	

Panel B. sensitivity subset (randomized short-course SPRING-01 subset)				
Group	pCR, n (%)	non-pCR, n (%)	Total, n	P value
Low-risk				0.794
nCRT	12 (70.5)	5 (29.5)	17	
nICRT	19 (79.2)	5 (20.8)	24	
High-risk				0.219
nCRT	4 (16.0)	21 (84.0)	25	
nICRT	7 (36.8)	12 (63.2)	19	

Data are presented as n (%), unless otherwise specified. The MRI-based risk score ranges from 0 to 3 points (one point each for mrEMVI positivity, mrMRF involvement, and tumor length ≥ 5 cm). Low-risk was defined as 0–1 points and high-risk as 2–3 points. P values were calculated using Fisher’s exact test to compare pCR rates between nCRT and nICRT within each risk category. nCRT, neoadjuvant chemoradiotherapy; nICRT, neoadjuvant immunotherapy plus chemoradiotherapy; pCR, pathological complete response.

To reduce the influence of treatment heterogeneity, we repeated the subgroup analysis in the randomized short-course SPRING-01 subset. Similar findings were observed in the sensitivity subset (**Table 3B**). Among low-risk patients, pCR rates were 79.2% (19/24) with nICRT and 70.6% (12/17) with nCRT (P = 0.794). In the high-risk subgroup, pCR rates were 36.8% (7/19) and 16.0% (4/25), respectively (P = 0.219). Although statistical significance was not retained in the subset analysis, likely owing to limited sample size, the numerically larger pCR benefit in MRI-defined high-risk patients remained evident.

### 3.5 Subgroup analysis

**Figure 3:**
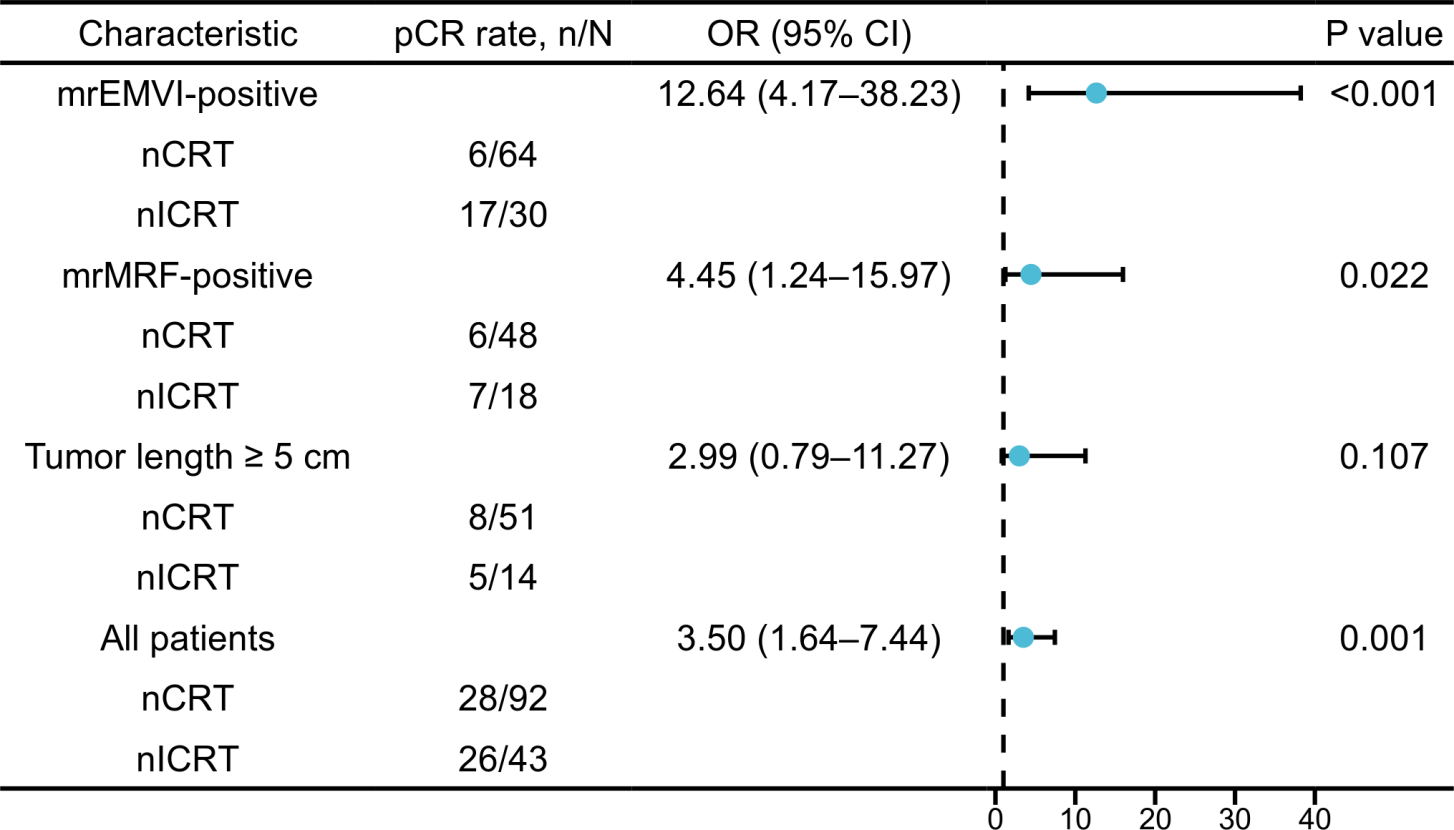
Forest plot of odds ratios for pathological complete response (pCR) with nICRT versus nCRT in predefined subgroups. Points indicate odds ratios (ORs), and horizontal bars indicate 95% confidence intervals (CIs). The vertical dashed line indicates OR = 1.0. ORs > 1 indicate higher odds of pCR with nICRT than with nCRT. ORs, 95% CIs, and Wald test P values were estimated using univariable logistic regression with nCRT as the reference group. nCRT, neoadjuvant chemoradiotherapy; nICRT, neoadjuvant immunotherapy plus chemoradiotherapy; mrEMVI, magnetic resonance imaging-detected extramural venous invasion; mrMRF, magnetic resonance imaging-detected mesorectal fascia involvement.

In predefined subgroup analyses, the association between treatment group and pCR was assessed using univariable logistic regression, with nCRT as the reference category (**Figure 3**). Overall, nICRT was associated with significantly higher odds of achieving pCR than nCRT (OR, 3.50; 95% CI, 1.64–7.44; P = 0.001). This association was particularly evident in patients with mrEMVI-positive tumors (OR, 12.64; 95% CI, 4.17–38.23; P < 0.001) and mrMRF-positive tumors (OR, 4.45; 95% CI, 1.24–15.97; P = 0.022). In the subgroup with tumor length ≥ 5 cm, nICRT also showed a numerically higher pCR rate, although the difference did not reach statistical significance (OR, 2.99; 95% CI, 0.79–11.27; P = 0.107).

### 3.6 ROC analysis

**Figure 4:**
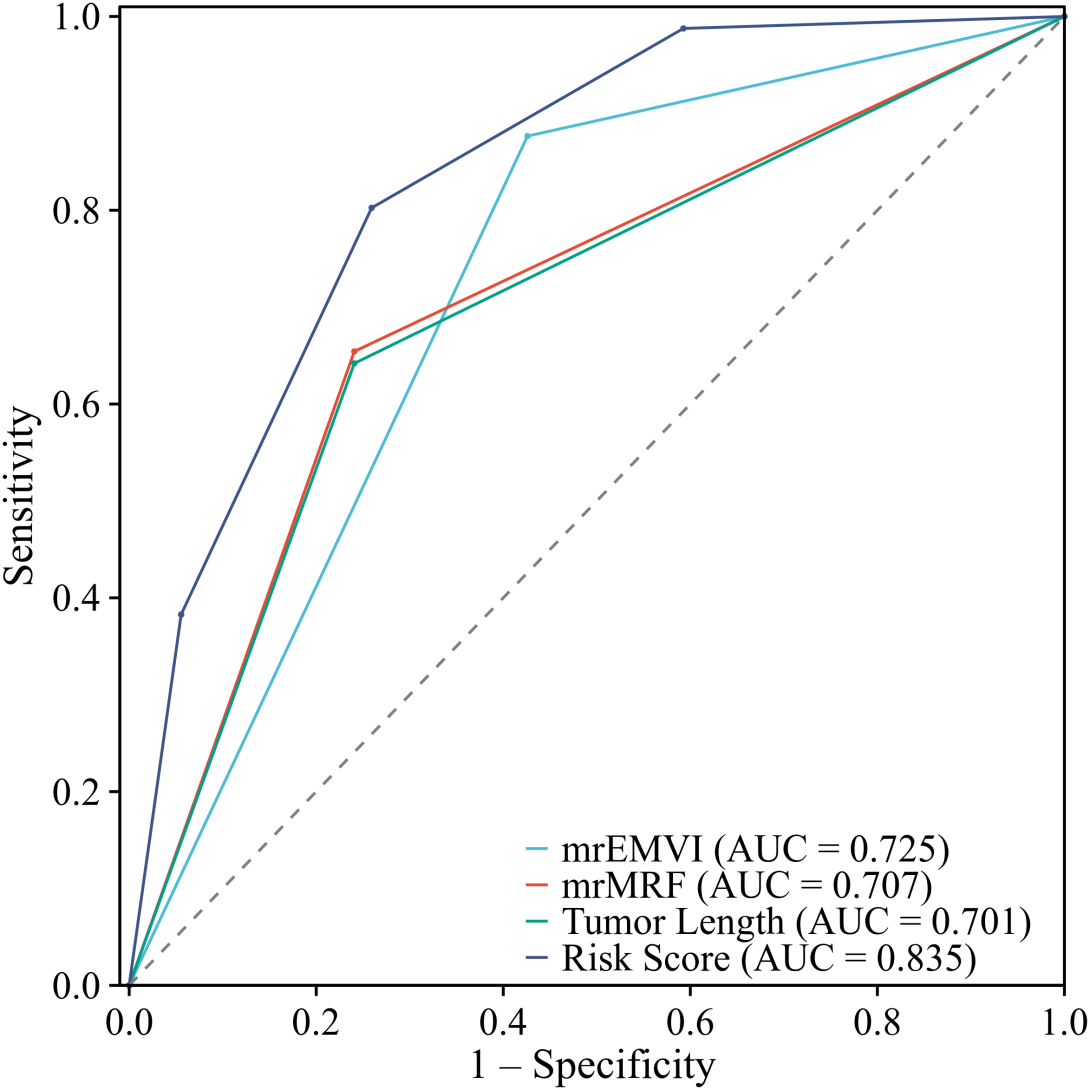
Receiver operating characteristic (ROC) curves for predicting pathological complete response (pCR) using individual MRI features and the composite MRI-based risk score. The diagonal dashed line represents no discriminative ability (AUC = 0.5). The AUCs were 0.725 for mrEMVI, 0.707 for mrMRF, 0.701 for tumor length ≥ 5 cm, and 0.835 for the MRI-based risk score. AUC, area under the receiver operating characteristic curve; mrEMVI, magnetic resonance imaging-detected extramural venous invasion; mrMRF, magnetic resonance imaging-detected mesorectal fascia involvement.

The MRI-based risk stratification system combining mrEMVI, mrMRF, and tumor length ≥ 5 cm yielded an AUC of 0.835 (95% CI, 0.767–0.902), indicating good discrimination between patients with and without pCR (**Figure 4**). Internal validation using 2,000 bootstrap resamples showed minimal optimism, with an optimism-corrected AUC of 0.835. The optimism-corrected calibration slope and intercept were 0.999 and -0.003, respectively, indicating good calibration. Among individual MRI variables, mrEMVI showed the highest predictive performance (AUC, 0.725), followed by mrMRF (AUC, 0.707) and tumor length (AUC, 0.701). The composite score significantly outperformed each single parameter (all P < 0.05; **Table 4**). Detailed bootstrap validation results are presented in **Supplementary Table S5**.

**Table 4:** Discriminative performance (AUC) of individual MRI predictors and the composite MRI-based risk score for predicting pCR.

Predictor	AUC	95% CI	P value
mrEMVI positivity	0.725	0.650–0.801	< 0.001
mrMRF involvement	0.707	0.629–0.784	< 0.001
Tumor length ≥ 5 cm	0.701	0.623–0.779	< 0.001
Risk Score	0.835	0.767–0.902	< 0.001

P values test whether the AUC differs from 0.50 (no discrimination) using the DeLong method. AUC, area under the receiver operating characteristic curve; CI, confidence interval; pCR, pathological complete response; mrEMVI, magnetic resonance imaging-detected extramural venous invasion; mrMRF, magnetic resonance imaging-detected mesorectal fascia involvement.

## 4 Discussion

In this retrospective study, we demonstrated that three pretreatment MRI features—mrEMVI positivity, mrMRF involvement, and tumor length ≥ 5 cm—were independently associated with a lower likelihood of achieving pCR following neoadjuvant therapy. A composite MRI risk score incorporating these three factors showed better discriminative performance than any individual feature, with satisfactory internal validity. Moreover, nICRT was associated with a higher pCR rate than nCRT in MRI-defined high-risk patients, whereas no clear additional benefit was observed in the low-risk group. These findings suggest that MRI-based risk stratification may help identify clinically relevant subgroups with different response patterns to intensified neoadjuvant treatment. Importantly, the results should be interpreted as exploratory rather than practice-changing.

Several pretreatment MRI features have previously been reported as predictors of tumor response in LARC. For example, a dynamic nomogram incorporating CEA, baseline T/N stage, and mrEMVI was developed for pCR prediction (20). Zhang et al. identified cN0–1 disease, smaller tumor size, and lower CEA level as factors associated with favorable regression (21), whereas Erozkan et al. found that lower tumor location, absence of EMVI, and absence of MRF involvement predicted complete response (22). Consistent with these studies, our analysis identified mrEMVI positivity, mrMRF involvement, and tumor length ≥ 5 cm as independent predictors of non-pCR. Collectively, these features may reflect greater local invasiveness, vascular involvement, and tumor burden, all of which are biologically plausible indicators of reduced responsiveness to neoadjuvant treatment.

The predictive value of each individual MRI parameter in our cohort was only moderate, supporting the rationale for a composite scoring approach. Compared with radiomics-based models that rely on extraction of numerous high-dimensional imaging features, our three-factor score is based on dichotomized variables that are routinely assessable in standard rectal MRI interpretation, which enhances interpretability and potential clinical usability. The composite score achieved an AUC of 0.835 (95% CI, 0.767–0.902), significantly outperforming each individual MRI feature. Although the score was developed and tested in the same cohort, bootstrap internal validation suggested minimal optimism, supporting its stability within this dataset. Nevertheless, external validation remains necessary before broader application.

A key finding of this study was that the association between nICRT and improved pCR rates appeared more evident in the MRI-defined high-risk subgroup, with no significant difference observed in low-risk patients. This finding is clinically relevant because patients with positive mrEMVI, involved mrMRF, or long-segment tumors generally represent a population with more aggressive local disease and a lower probability of pCR with standard treatment (18, 23, 24). However, this pattern should not be interpreted as direct evidence that MRI-defined high-risk tumors are intrinsically more responsive to immunotherapy. The nICRT regimen in our study differed from conventional nCRT not only by the addition of sintilimab but also by overall treatment composition and intensity. Therefore, the higher pCR rate observed in high-risk patients may reflect, at least in part, the effects of treatment intensification and multimodal synergy rather than a purely immunotherapy-specific effect.

This distinction is particularly important in light of the molecular background of rectal cancer. Most rectal cancers are pMMR/MSS, and immunotherapy in unselected rectal cancer remains investigational rather than standard of care (6). In our cohort, most patients with available molecular data were pMMR, and only a very small number of dMMR tumors were identified. Thus, our results should be interpreted cautiously. At the same time, combined chemoradiotherapy and immunotherapy may still show biological activity in selected pMMR/MSS tumors. Mechanistically, chemoradiotherapy can remodel the tumor microenvironment by inducing immunogenic cell death, upregulating PD-L1, and increasing T-cell infiltration, which may sensitize pMMR/MSS tumors to combined immunotherapy (9, 25). In this context, MRI-defined high-risk features may reflect biologically aggressive tumors in which intensified multimodal treatment, including immunotherapy-containing regimens, has greater incremental activity. This interpretation is broadly consistent with emerging prospective evidence from the UNION study (26), in which neoadjuvant short-course radiotherapy followed by camrelizumab and chemotherapy produced encouraging pCR rates in clinically challenging subgroups, including patients with high-risk baseline features such as cT4/cN2 disease, EMVI positivity, or MRF involvement. However, these imaging features should not be regarded as direct biomarkers of immune responsiveness. Prior studies have suggested that adverse features such as EMVI and greater tumor burden may be linked to distinct immune-related states (17, 18, 27), but these possible biological explanations were not assessed in our cohort and therefore remain speculative, requiring further validation in studies integrating MRI with molecular and immune profiling.

To reduce the influence of treatment heterogeneity, we performed a sensitivity analysis restricted to the randomized short-course SPRING-01 subset. The direction of the association remained similar to that in the full cohort, but statistical significance was no longer observed, most likely because of the smaller sample size and limited statistical power. This finding reinforces that our subgroup results should be considered hypothesis-generating and interpreted with caution. In the absence of integrated immune profiling, the precise immunological mechanisms remain unclear. Prospective studies with integrated immune and molecular correlative analyses are warranted to validate these findings.

The predefined subgroup analyses further suggested that the association between nICRT and higher pCR rates was most evident in patients with mrEMVI-positive or mrMRF-involved tumors, whereas no statistically significant difference was observed in patients defined only by tumor length ≥ 5 cm. EMVI and MRF involvement are established adverse features reflecting vascular invasion and advanced local extension (14), respectively, and may therefore capture a more aggressive disease phenotype than tumor bulk alone. Some prior molecular studies have suggested that EMVI-positive tumors may be linked to distinct immune-related features (27); however, these observations were not assessed in our cohort and should be interpreted cautiously. Likewise, greater tumor burden has been linked in some studies to tumor microenvironmental features that may influence treatment response (28, 29), although the relevance of these observations to MRI-defined tumor length in our analysis remains indirect. By contrast, tumor length primarily reflects tumor size and may be a less specific indicator of differential treatment response. Tumor length was dichotomized at 5 cm based on thresholds used in previous studies (18, 19). Variation in the cutoffs applied across studies may partly explain the inconsistent prognostic value of tumor size reported in the literature. However, these subgroup findings remain exploratory. Because subgroup sample sizes were limited and no formal interaction analysis was performed, they should not be overinterpreted as definitive evidence of treatment-effect heterogeneity.

Our study also examined pretreatment blood-based markers, including CEA, platelet count, and inflammatory indices (NLR, PLR, LMR), but none showed a consistent independent association with pCR. This differs from prior reports in which CEA, platelet count, or composite inflammatory scores were incorporated into predictive models for response to nCRT (18, 30–34). In our cohort, platelet count was related to pCR only in univariable analysis, similar to findings by Policicchio et al. (33). The lack of significance for CEA and inflammatory indices may reflect their biological non-specificity and susceptibility to confounding factors. Therefore, these variables were not retained in the final model.

This study has several limitations. First, it was a single-center retrospective analysis with a relatively small sample size, and the findings lack external validation. No formal *a priori* sample size calculation was performed; therefore, all subgroup findings should be regarded as exploratory. Second, the nCRT group included both short-course radiotherapy plus consolidation chemotherapy and long-course chemoradiotherapy, introducing treatment heterogeneity. Although the sensitivity analysis in the randomized short-course subset showed a directionally similar pattern, this heterogeneity remains an important structural limitation of the study design. Third, MMR status was unavailable for a subset of patients, and the small number of dMMR cases precluded meaningful analyses stratified by MMR status. Thus, we cannot exclude residual confounding related to molecular subtype. Fourth, the study did not include immune microenvironment characterization or in-depth molecular profiling. Without data on tumor-infiltrating lymphocytes, PD-L1 expression, immune gene signatures, or spatial histopathologic correlates, we cannot determine whether the observed pCR differences reflect a true immunotherapy-specific effect or simply the consequences of treatment intensification. Moreover, pCR was the sole clinical endpoint. Although clinically relevant, pCR cannot substitute for long-term oncological outcomes and may not fully capture the complex immune-related response patterns associated with immunotherapy-containing regimens. Finally, the proposed MRI-based score was evaluated in the context of specific neoadjuvant regimens, and its generalizability to other treatment protocols remains uncertain. In addition, follow-up duration was insufficient to assess long-term oncological outcomes.

In conclusion, a simple composite MRI risk score based on mrEMVI, mrMRF, and tumor length effectively stratifies LARC patients by pCR likelihood and identifies a high-risk subgroup that appears to derive greater benefit from neoadjuvant immunotherapy plus chemoradiotherapy. These exploratory findings warrant external validation and prospective confirmation in trials incorporating MRI-based stratification and immune microenvironment profiling.

## Data Availability

The raw data supporting the conclusions of this article will be made available by the authors, without undue reservation.
