# Supplementary material for: MRI-guided risk stratification for neoadjuvant immunotherapy in rectal cancer
Source: Front Immunol. 2026 May 29;17:1782231. doi: 10.3389/fimmu.2026.1782231 (PMC13260345; doi:10.3389/fimmu.2026.1782231)
Supplement: Supplementary file 1 [file DataSheet1.docx]

Supplementary Table S1. Representative Protocols for the MR imaging sequence

| Sequence | TR (ms) | TE (ms) | FOV (mm) | Matrix | NEX | Slice thickness (mm) | Intersection gap (mm) |
| --- | --- | --- | --- | --- | --- | --- | --- |
| Oblique axial T2WI | 3000 | 90 | 220 | 256×320 | 2 | 3 | 0 |
| Sagittal T2WI | 3000 | 90 | 220 | 256×320 | 2 | 3 | 0.3 |
| Coronal T2WI | 3000 | 90 | 220 | 256×320 | 2 | 3 | 0.3 |
| Axial T1WI | 450 | 10 | 220 | 256×192 | 2 | 3 | 0.3 |
| DWI (b values 0/1000 (s/mm²) | 4000 | 82 | 400 | 128×160 | 3 | 5 | 1 |
| ADC map | 4000 | 82 | 400 | 128×160 | 3 | 5 | 1 |

Note—TR = repetition time; TE = echo time; FOV = field of view; NEX = number of excitations; T2WI = T2-weighted imaging; T1WI = T1-weighted imaging; DWI = diffusion-weighted imaging; ADC = apparent diffusion coefficient.

Supplementary Table S2. Comparison of clinicopathological, laboratory, and MRI variables by pCR status

| Variable | pCR (n = 54) | non-pCR (n = 81) | P Value |
| --- | --- | --- | --- |
| Sex |  |  | 0.649 |
| Female | 18/54 (33.3) | 24/81 (29.6) |  |
| Male | 36/54 (66.7) | 57/81 (70.4) |  |
| Age (years) |  |  | 0.573 |
| < 60 | 24/54 (44.4) | 40/81 (49.4) |  |
| ≥ 60 | 30/54 (55.6) | 41/81 (50.6) |  |
| mrT stage |  |  | 0.375 |
| T2 | 1/54 (1.9) | 0/81 (0.0) |  |
| T3 | 37/54 (68.5) | 52/81 (64.2) |  |
| T4 | 16/54 (29.6) | 29/81 (35.8) |  |
| mrN stage |  |  | 0.637 |
| N0 | 4/54 (7.4) | 6/81 (7.4) |  |
| N1 | 27/54 (50.0) | 34/81 (42.0) |  |
| N2 | 23/54 (42.6) | 41/81 (50.6) |  |
| mrEMVI status |  |  | < 0.001 |
| Negative | 31/54 (57.4) | 10/81 (12.3) |  |
| Positive | 23/54 (42.6) | 71/81 (87.7) |  |
| mrMRF involvement |  |  | < 0.001 |
| Negative | 41/54 (75.9) | 28/81 (34.6) |  |
| Positive | 13/54 (24.1) | 53/81 (65.4) |  |
| Tumor length (cm) |  |  | < 0.001 |
| < 5 | 41/54 (75.9) | 29/81 (35.8) |  |
| ≥ 5 | 13/54 (24.1) | 52/81 (64.2) |  |
| Distance to the anal verge (cm) |  |  | 0.053 |
| ≤ 5 | 27/54 (50.0) | 27/81 (33.3) |  |
| > 5 | 27/54 (50.0) | 54/81 (66.7) |  |
| Tumor circumferential extent |  |  | 0.919 |
| < 1/4 | 1/54 (1.9) | 2/81 (2.5) |  |
| 1/4 ≤ x < 1/2 | 5/54 (9.3) | 5/81 (6.2) |  |
| 1/2 ≤ x < 3/4 | 10/54 (18.5) | 15/81 (18.5) |  |
| 3/4 ≤ x ≤ 1 | 38/54 (70.4) | 59/81 (72.8) |  |
| Maximum tumor thickness (cm) |  |  | 0.7 |
| < 1 | 6/54 (11.1) | 12/81 (14.8) |  |
| 1 ≤ X < 2 | 44/54 (81.5) | 61/81 (75.3) |  |
| ≥ 2 | 4/54 (7.4) | 8/81 (9.9) |  |
| CEA (ng/mL) |  |  | 1.000 |
| < 5 | 32/54 (59.3) | 48/81 (59.3) |  |
| ≥ 5 | 22/54 (40.7) | 33/81 (40.7) |  |
| Type of operation |  |  | 0.257 |
| Dixon | 33/54 (61.1) | 60/81 (74.1) |  |
| Miles | 17/54 (31.5) | 18/81 (22.2) |  |
| Hartmann | 4/54 (7.4) | 3/81 (3.7) |  |
| PLT (×10^9/L) | 219.5 ± 69.3 | 261.2 ± 84.1 | 0.003 |
| NLR | 2.32 (1.64–3.42) | 2.20 (1.61–2.99) | 0.374 |
| PLR | 155.77 (109.30–210.21) | 159.55 (128.12–224.72) | 0.503 |
| LMR | 3.82 (2.65–5.67) | 3.82 (3.03–4.69) | 0.713 |

Note—Data are presented as n/N (%) for categorical variables and as mean ± standard deviation or median (interquartile range), as appropriate. P values were calculated using the chi-square test or Fisher’s exact test for categorical variables and the Student’s t-test or Mann–Whitney U test for continuous variables, as appropriate (two-sided). pCR = pathological complete response; mrT = MRI-based T stage; mrN = MRI-based N stage; mrEMVI = magnetic resonance imaging-detected extramural venous invasion; mrMRF = magnetic resonance imaging-detected mesorectal fascia involvement; CEA = carcinoembryonic antigen; PLT = platelet count; NLR = neutrophil-to-lymphocyte ratio; PLR = platelet-to-lymphocyte ratio; LMR = lymphocyte-to-monocyte ratio.Data are presented as n/N (%) for categorical variables and as mean ± standard deviation or median (interquartile range), as appropriate. P values were calculated using the chi-square test or Fisher’s exact test for categorical variables and the Student’s t-test or Mann–Whitney U test for continuous variables, as appropriate (two-sided). pCR = pathological complete response; mrT = MRI-based T stage; mrN = MRI-based N stage; mrEMVI = magnetic resonance imaging-detected extramural venous invasion; mrMRF = magnetic resonance imaging-detected mesorectal fascia involvement; CEA = carcinoembryonic antigen; PLT = platelet count; NLR = neutrophil-to-lymphocyte ratio; PLR = platelet-to-lymphocyte ratio; LMR = lymphocyte-to-monocyte ratio.

Supplementary Table S3. Interobserver agreement (κ) for MRI risk factors

| Variables | κ Value | 95% CI | *P* Value |
| --- | --- | --- | --- |
| mrEMVI | 0.787 | 0.67–0.90 | < 0.001 |
| mrMRF | 0.733 | 0.62–0.85 | < 0.001 |
| Tumor length ≥ 5cm | 0.881 | 0.80–0.96 | < 0.001 |

Note—Data are presented as κ (kappa) values with corresponding 95% confidence intervals (CIs). mrEMVI = magnetic resonance imaging-detected extramural venous invasion; mrMRF = magnetic resonance imaging-detected mesorectal fascia involvement.

Supplementary Table S4. Discrimination of the MRI-based risk score for pCR within each treatment group

| Group | pCR, n (%) | non-pCR, n (%) | Total, n | P Value |
| --- | --- | --- | --- | --- |
| nCRT |  |  |  | 0.000 |
| Low-risk | 21 (65.6) | 11 (34.4) | 32 |  |
| High-risk | 7 (11.7) | 53 (88.3) | 60 |  |
| nICRT |  |  |  | 0.005 |
| Low-risk | 19 (79.2) | 5 (20.8) | 24 |  |
| High-risk | 7 (36.8) | 12 (63.2) | 19 |  |

Note—Data are presented as n (%), unless otherwise specified. Low-risk was defined as 0–1 points and high-risk as 2–3 points in the MRI-based risk score. P values were calculated using Fisher’s exact test to compare pCR rates between low-risk and high-risk groups within each treatment group. nCRT = neoadjuvant chemoradiotherapy; nICRT = neoadjuvant immunotherapy plus chemoradiotherapy; pCR = pathological complete response.

Supplementary Table S5. Bootstrapped internal validation of the MRI-based risk score for predicting pCR

| **Metric** | **Value** |
| --- | --- |
| Sample size | 135 |
| Apparent AUC | 0.835 |
| 95% CI for apparent AUC | 0.767–0.902 |
| Number of bootstrap resamples | 2000 |
| Optimism-corrected AUC | 0.835 |
| Optimism-corrected calibration slope | 0.999 |
| Optimism-corrected calibration intercept | -0.003 |
| Mean absolute error | 0.041 |
| Mean squared error | 0.00245 |
| 0.9 quantile of absolute error | 0.073 |

Note—Internal validation was performed using bootstrap resampling (2,000 resamples). Apparent performance was calculated in the original sample; optimism-corrected estimates were obtained after bootstrap adjustment. AUC = area under the receiver operating characteristic curve; CI = confidence interval; pCR = pathological complete response.
